# Bevacizumab combined with chemotherapy and anti-PD-1 therapy for the first-line treatment of previously untreated metastatic triple-negative breast cancer

**DOI:** 10.1136/jitc-2025-012312

**Published:** 2025-04-30

**Authors:** Bi-Cheng Wang

**Affiliations:** 1 Cancer Center, Union Hospital, Tongji Medical College, Huazhong University of Science and Technology, Wuhan, Hubei, China

**Keywords:** Breast Cancer, Chemotherapy, Immunotherapy

Meiting Chen and colleagues conducted a study to assess the efficacy and safety of a first-line treatment regimen combining bevacizumab, nab-paclitaxel, and the anti-programmed death 1 antibody tislelizumab in patients with previously untreated metastatic triple-negative breast cancer (TNBC).^1^ While the study offers valuable preliminary insights into this combinatorial approach, several notable limitations and methodological concerns raise questions regarding the robustness and generalizability of the findings.

First, although the study was registered in the Chinese Clinical Trial Registry on April 11, 2022, patient enrollment began much earlier, spanning from March 11, 2021, to February 5, 2024. Notably, the ethics committee approved the study on September 28, 2021, yet the first participant was enrolled prior to both ethical approval and trial registration. Initiating patient enrollment before formal trial registration undermines the principles of transparency and prospective trial design and is generally considered inappropriate in clinical research.

Second, the study was designed as a parallel, phase II trial in which patients were randomly assigned to receive either 7.5 mg/kg (group 1) or 15 mg/kg (group 2) of bevacizumab. However, the authors did not specify the randomization algorithm or method used, nor did they report any stratification factors. Given the relatively small sample size and apparent baseline imbalances between groups, the validity of subgroup comparisons is questionable and subject to considerable bias. Furthermore, some comparisons appear to lack clinical relevance; for instance, patients with liver metastases are already known to have poorer outcomes compared with those without, as previously reported.^2^

Third, the rationale for selecting objective response rate as the primary endpoint warrants further clarification, particularly given that progression-free survival (PFS) or overall survival (OS) might be more appropriate based on the study’s reported findings. Moreover, although the duration of response was listed as a secondary endpoint, no corresponding data were presented, limiting the interpretability of the reported treatment efficacy.

Fourth, although programmed death ligand 1 (PD-L1) testing was not mandated by the study protocol, approximately 36.7% of enrolled patients underwent assessment. In both univariate and multivariate analyses of OS and PFS, no significant differences were observed between PD-L1-positive and PD-L1-negative groups. However, these negative findings were not clearly explored or discussed. Given the results from the KEYNOTE-355 trial, which demonstrated enhanced efficacy of pembrolizumab plus chemotherapy with increasing PD-L1 expression in previously untreated locally recurrent inoperable or metastatic TNBC,^3^ it would be important to complete PD-L1 assessment in the remaining patients and conduct subgroup analyses based on well-balanced baseline characteristics.

Fifth, in the reported subgroup of PD-L1-positive patients, the combination of bevacizumab, tislelizumab, and nab-paclitaxel did not appear to improve median OS (19.6 vs 23.0 months) or median PFS (7.8 vs 9.7 months) when indirectly compared with the KEYNOTE-355 trial results.^3 4^ These findings raise questions regarding the added value of bevacizumab in the first-line treatment of previously untreated TNBC and warrant further investigation.

Sixth, minor inconsistencies and editorial errors in the article may affect clarity and should be addressed. For example, contradictory descriptions of the PD-L1-negative population are present in different sections: the Highlights state that ‘PD-L1-negative patients…constitute approximately 70% of the population,’ while the Introduction section claims that ‘approximately 70% of PD-L1-negative patients do not benefit from the addition of immunotherapy’. Additionally, “online supplemental figure 4” is cited in reference to both the nomograms and the swimming plot based on PD-L1 expression, which may cause confusion. More rigorous and consistent reporting would help strengthen the credibility of the study.

In conclusion, it is crucial to acknowledge the limitations of this study to ensure the appropriate interpretation and application of its findings in the treatment of patients with previously untreated metastatic TNBC.
